# CMOS compatible novel integration solution for broad range tunable photodetection using phase-change material based heterostructures

**DOI:** 10.1038/s41598-020-67950-2

**Published:** 2020-07-07

**Authors:** Vibhu Srivastava, Prateek Mishra, Sunny

**Affiliations:** Department of Electronics and Communication Engineering, Indian Institute of Information Technology Allahabad, Prayagraj, 211015 India

**Keywords:** Optoelectronic devices and components, Photonic devices

## Abstract

Heterostructures (HS) have always been in attraction due to their inherited properties and different important applications. Integration of a phase-change material (PCM) with HS can tremendously extend the operating and application range using the “phase-tuning” of PCM for any optoelectronic devices. In the present study, we report a detailed study of electrical and optoelectronic characteristics of a p-p and p-n HS combining Ge_2_Sb_2_Te_5_ (GST) and Si. Reasonable 2 order of resistance switching is achieved by thermal annealing. The changes in optical properties are analysed using Ellipsometry, UV–Vis–NIR and Raman spectroscopy to speculate the optoelectronic behaviour of GST/Si samples. The optical and electrical characterization were analysed with aluminium (Al), platinum (Pt) and Ti/Au contacts. Appreciable rectifications varying from 500 to 1,000 at lower voltages are achieved with different contacts for both phases of GST. The change in rectification amount and current polarity are obtained with different kinds of contacts and at different incident wavelengths indicating different mechanisms of charge separation and collection. Responsivity of more than 9 A/W with < 1,000 photo-current to dark-current ratio is demonstrated in wavelength range of 0.8–2 μm under moderate range of biasing under ~ μW source power illumination. The characteristics obtained were justified with the prediction of band alignment with the help of work-function difference measurement by Kelvin-probe force microscopy and carrier density measurement by Hall experiment. Our results provide understanding to the opto-electrical behaviour of a heterojunction made of stacking PCM (GST) on Si highlighting their future use in photonic/optoelectronic-integrated circuits.

## Introduction

Heterostructures have received intensive attentions due to their excellent intrinsic properties and wide applications ever since their emergence at very dawn of semiconductor (SC) technology in 1932^1^. Heterostructures (HS), especially heterojunctions, juxtapose two different layers of materials which usually are of different materials having different properties. If the two materials are of same type (n-n or p-p) then it is called as “isotype” otherwise known as “anisotype” (*e.g.* n-p type)^2^. Both types of the HS have their different potential applications^3^. Fundamentally, instead of controlling the electrical parameters of a semiconductor by external doping and carrier injection, heterostructures can inherit different material properties of two layers *e.g.* controlling the charge transfer at the interface and controlling the fundamental parameters such as bandgap, effective masses, mobilities, refractive indices, etc., in the semiconductor based devices^4^. Nowadays, scientists/researchers are more interested in the interactions at the junction interface to improve the performance and sometimes synergistic properties as well^5^. HS are widely used as the basic building blocks in conventional devices such as photodiodes^6^, solar cells^7^, LEDs^8^, as well as in complicated structures like double-HS (DHS) LASERs^9^, HEMTs^10^, Quantum-well^11^ LASER diodes^12^ etc.

It is well known that the traditional junction-less devices or normal p-n junctions or Metal-SC based HSs suffer from large dark currents and is much prone to external stimuli^13^. This limits their use and degrades the effectiveness in highly sensitive systems. This also extends the researcher’s effort to develop more complex structures, like quantum-well/dot, which are difficult to grow and to operate at normal conditions. This might get worsen if a compound SC technology is involved in the processing^14^. Si/Ge/SiGe based HSs gained popularity due to their higher compatibility with Si-based technology^15^. However, they are limited to fulfil the wide range of applications, even have the bottleneck in performance in a particular application specially limiting the operating bandwidth in optoelectronic devices.

Nowadays, compound materials are being used to make high performance HS based simpler devices, like photodetectors^16^ and solar cells^17^. To extend the limits of the devices, electronically tunable bandgap materials are being used^18–20^. This bandgap tuning can be only achieved by thin layered materials, hence, 2D materials with other bulk materials making a HS came into existence^21,22^. The formation of Van-der Walls type bonding and layering/stacking have been widely adopted to tune the properties of 2D materials^23,24^. Many metal (specifically transition metals) oxides, halides and chalcogenides (specially transition-metal-dichalcogenides (TMD)) are under investigation and providing great outcomes, like good responsivity, detectivity, low dark-current, low noise, higher sensitivity, etc.^25–27^. It is worth to note here that tuning is achieved by changing the number of layers and interface physically. This implies that a particular device made up of some number of layers can be used for a particular operation and the properties become static. For other similar applications, such as broadband photodetector operating at multiple wavelengths and electronic/optical switch/modulators/limiters, another device has to be fabricated with different layers/stacks. In spite of great achievements, outcomes and technological advancement, 2D materials suffer huge drawbacks discussed ahead. Firstly, it is still a challenge to precisely control the number of layers. Second, the physical and electrical stacking faults are common in 2D materials due to irregular energy barrier in layers producing stress/strain in-between layers and distorted energy bands^28^. Thirdly, 2D layered materials encounters with gross surface recombination which completely annihilate the purpose of HS^29^. Recently, Iqbal et al*.*^26^ showed that the electronic band structure of atomically thin 2D layers is not completely intrinsic to SC-substrate and can be strongly affected by the surrounding environment leading to abnormal electrical transport, and hence recommended bandgap renormalizations which further adds to the complexity.

Bulk materials such as GaAs, InGaAs, InGaAsP, etc.^30–32^, are also in tremendous use/production and many are under investigations depending upon application, compatibility, abundance, cost effectiveness, etc^33^. Bulk materials do not go through the above mentioned/discussed drawbacks of 2D layered materials. However, the idea of “tuning” is absent in the case of bulk materials. To include the “tuning” phenomenon in bulk, unlike “static” “tuning” in 2D material, the phase-change materials (PCMs) can be utilised to make the same device with similar processes/materials that can work for different applications by achieving different material properties in different phases. Such optoelectronic devices have been demonstrated using PCMs^34–38^. PCMs are the materials whose phase can be modulated or interchanged between amorphous to crystalline by applying some external stimuli, and remain in its stable phase until another external stimuli is applied^39,40^. Among lots of PCMs found till date, a chalcogen-based compound semiconductor PCM, Ge_2_Sb_2_Te_5_ (GST) is the most extensively explored and widely used in numerous applications^41^. Also, considering the optical fibre communication systems working at 1.55 μm wavelength^42^ and optoelectronic devices working in near-infra red (NIR) range^43^, GST would be most suitable candidate. Amorphous GST (AGST) having bandgap around 0.65 eV^44,45^ can be changed into its crystalline (CGST) form by thermal, optical and electrical excitation^46^ providing lesser bandgap of around 0.5 eV^44,47^. The crystalline phase further can be changed to amorphous with same stimuli under specific condition^46,48^. GST provides very distinct material properties, such as mobility, resistivity, effective masses, refractive indices, etc., in both phases, making this material tunable and have been demonstrating a switch^49,50^, modulators^51^, phase-change memories^39^, photodetectors^52,53^, optical limiters^54^, transistors^55^, meta-materials^56^, etc. GST is a p-type material in its both phases and have monolithic crystal structure while above the transition temperature it exhibit rutile/FCC/hex structure^46^.

All optical fibre communication systems and its associated optical devices (LED and LASERs as source, photodetectors at receiver, switches, modulators, couplers and splitters etc.) are mostly based on Si technology. Combining the layers of GST with Si could be a feasible route to fabricate high quality p-n/p-p HS by harnessing the advantages of both materials. GST material is chosen due to its well-studied compatibility with Si^57^ and its huge absorption in visible-NIR spectral range^58^. Also, the photo-generated carriers in Si arising from the absorption also contribute to the photo-current and hence increasing the performance parameters. Furthermore, the asymmetric band offset with Si valance-band (VB)/conduction-band (CB) will offer an efficient collection/rectification of charge carriers^59^ and constitute low reflective losses as the refractive indices of both are compatible^60^. Miller et al*.*^61^ have carried out the electro-optical characteristic of optically induced phase change material (O-PCM) integrated with Si only at 2 μm wavelength. They used VO_2_ and few variant of GST (GeSe, Ge_2_Sb_2_Se_4_Te_1_) for the detailed study of OPCM on Si and demonstrated a modulator using the same. Recently, Sarwat et al*.*^52^ have engineered Au/ITO/GST/Pt nanoscale optical photodetector working on visible wavelength of 637 nm.

In the present study, we report the electrical and optoelectronic characterization of a p-p and p-n HS combining GST (p-type and PCM for tunability) and Si (n-type for p-n HS and p-type for p-p HS). A good amount of tunable rectification, even at lower biasing, is achieved (500 to 1,000 at 1 V). The same device can be turned into a Schottky rectifier when annealed at higher temperature. The change in rectification amount and current polarity are investigated with different kind of contacts and at different incident wavelengths and power. On basis of different phenomenon, we stipulated that there are different mechanism of charge separation and collection. A good responsivity at the desired wavelength range is obtained (> 9 A/W in between 0.8 μm to 2 μm range at moderate optical power of 3.38 μW) with higher enough photo-current to dark-current ratio of < 1,000. We have also investigated the band-alignment experimentally to justify the opto-electric behaviour of the system.

The outlines of this paper are as follows. Section II contains the details of experimental and execution part. Section III presents the results and discussion. Section IV finally concludes the findings of this work.

## Experiment

**Figure 1:**
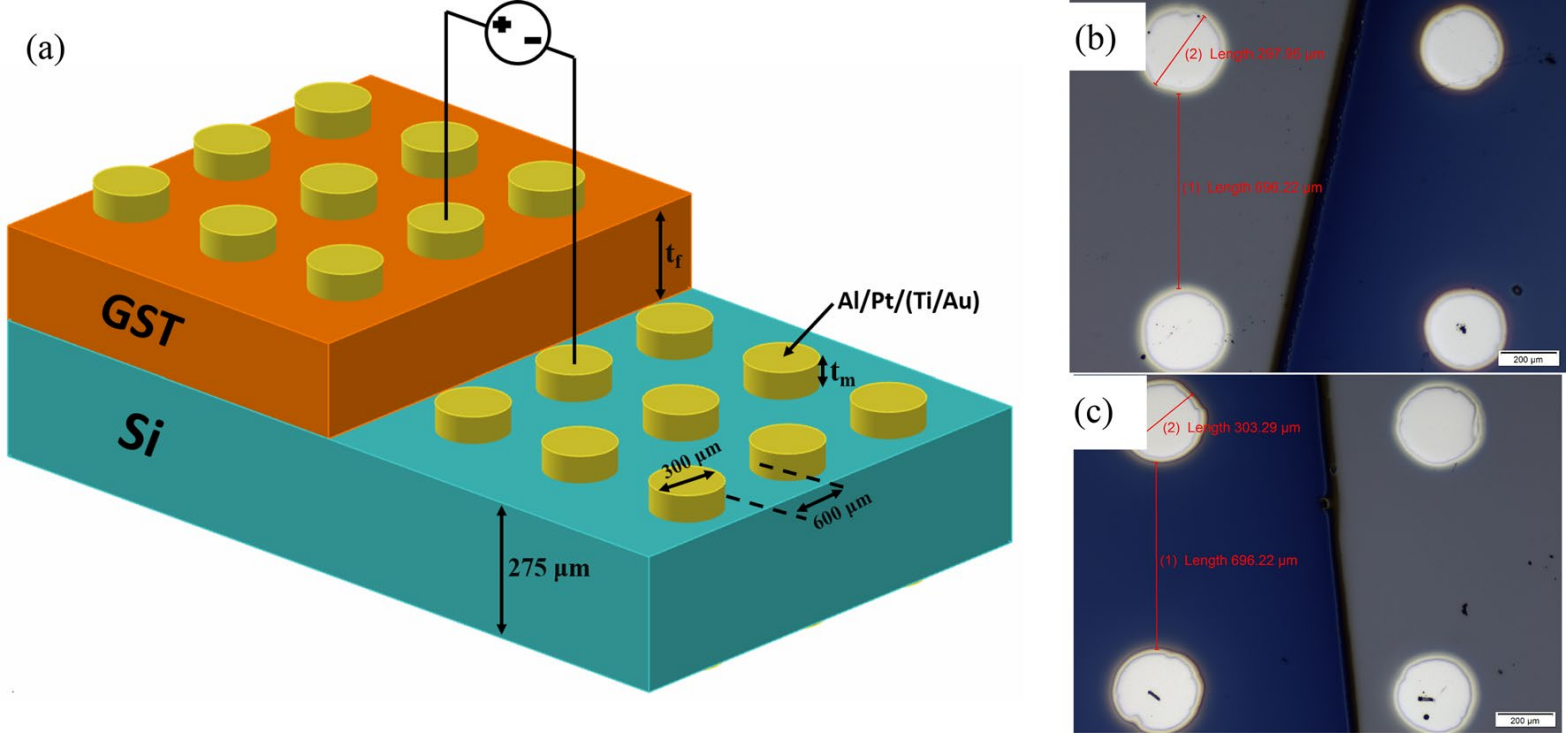
(**a**) Illustration of the fabricated device, (**b**, **c**) Optical images of the fabricated device.

The Si wafers are cleaned with RCA1 and RCA2 followed by dip in BHF to remove any residual oxides on the surface. To achieve lateral structure, only a certain part of Si is exposed to the deposition of GST material. GST material was sputtered using HHV 3.0A machine with a target (procured from American Elements, 99.9%) at base pressure of 2 × 10^–2^ Pa and high-vacuum pressure of 5 × 10^–5^ Pa in Ar environment for plasma at 5 sccm flow. The temperature of samples was not increased more than 45 °C during sputtering. Spectroscopic Ellipsometer (V. Vase, J. A. Woolam) is employed to measure the thicknesses. The optimization of thicknesses is listed elsewhere^62^. We have analysed the operation of fabricated device with three types of contacts: Aluminium deposited through thermal evaporation (HHV), Pt and Ti/Au are deposited through sputtering. The graphical representation of fabricated device is illustrated in Fig. 1a with all dimensions. Figure 1b,c are the microscopic images of real fabricated device taken form Olympus (BX53M) optical microscope.

For the thin film analysis, we use 100 nm as-deposited film *i.e.* AGST. The measurements of the refractive indices (*n*, *k*) were also obtained through spectroscopic Ellipsometer in the range of 800–2000 nm wavelength range at 75̊ of light incident angle. For this, Tauc-Lorentz model is employed for AGST while Cody–Lorentz + Oscillation model for CGST^47^ during measurement. For the crystallization, we used thermal heating that was done in a sealed chamber of N_2_ environment to avoid oxidation^63^ and excessive evaporation^47,64^ though there are little chances of occurrences of both, as no capping layer is used. The resistive switching of GST film depends on various factors, such as thickness of film, temperature, time of exposure, rate of heating and the type of substrate also^44^. As, in this work, the GST is deposited on another bulk Si which can affect the heat flow in the PCM and hence can manipulate the phase-change order as well as transition temperature. Differential scanning calorimetry (DSC) (STA8000 Perkin Elmer) has been employed to observe the heat flow in the combined structure. A moderate heating rate of 5 °C/min was chosen to effectively observe the heat-flow along with the switching^65^. While heating the samples, the atomic arrangement changes inside the sample to a higher and regular density crystal, usually to FCC or Hexagonal structure. Resistivity *vs.* temperature is given to elaborate the transition along with order of switching. The deposition of material and changes due to crystallization have been confirmed through XRD (Rigaku MiniFlex). SEM (JEOL JSM-7100F) was used to analyse the surface morphology. Compositional analysis was done through EDX (Oxford Ultim Max attached to SEM). For optical properties and to get the optical bandgap, UV–Vis–NIR analysis were done on both AGST and CGST in 800 nm to 2000 nm wavelength range (Shimadzu 3000XUV). Raman spectroscopy (Priceton Instruments Acton Spectra Pro 2500i) was used to analyse the emission spectra along with changes in the samples during crystallization. Kelvin probe force microscopy (KPFM) (Asylum Research from Oxford, Model: MFP3D, with Ti/Ir coated Si tip) were employed to measure the work-function/surface-potential difference at the Si/GST interfaces to estimate band alignments in combination with analysis obtained (carrier concentration) from Hall measurement (Lake Shore 8400 and Toyo Corporation). Keithley 4200SCS was used for electrical characterization. Light rays of different wavelengths incident on the samples were obtained through tunable light source NewPort TLS 300XU. During all electrical and optical I–V characterization, the samples were remaining probed at SemiProbe probing station through two 4,225 RPM/SMU.

## Results

**Figure 2:**
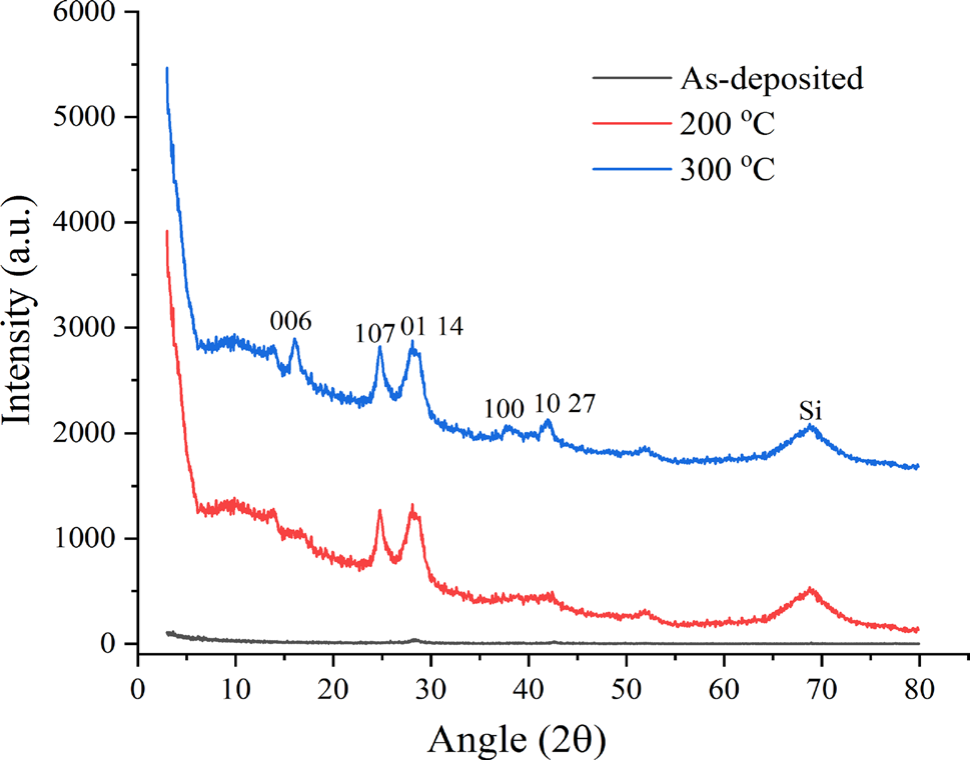
XRD spectra of the GST/Si films prepared and annealed at different temperatures.

**Figure 3:**
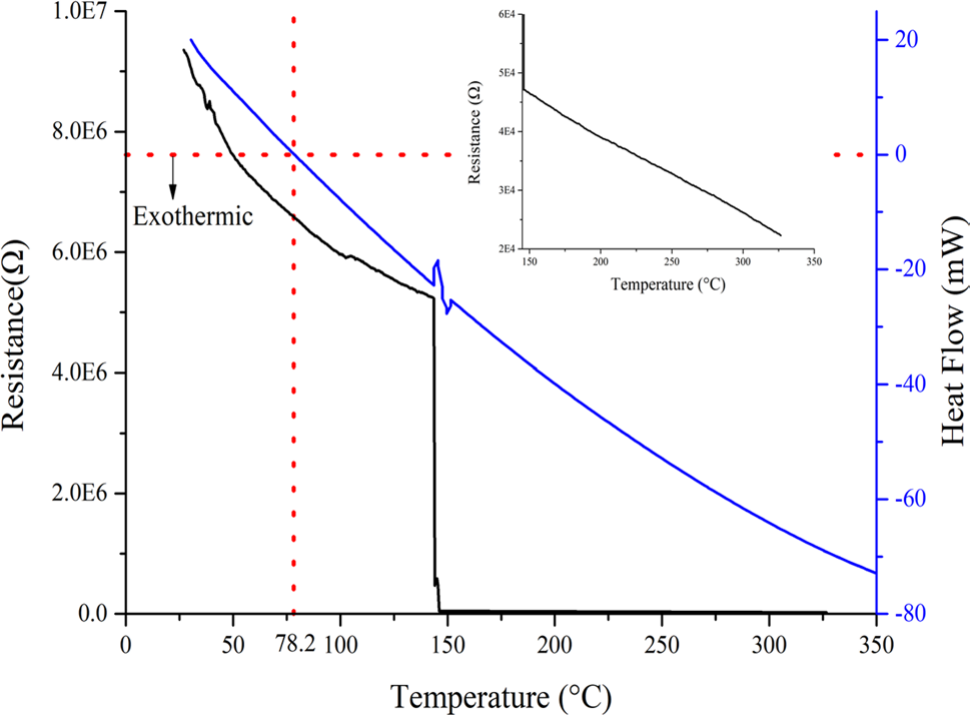
Thermal switching realization of the GST thin film along with DSC analysis. The resistivity curve at higher temperature is shown in inset.

The crystallization was achieved by heating of the GST/Si samples, during which a few of the upper layers may wear-off/disperse. The as-deposited layers of GST were optimized for 100 nm, whereas after heating for 15 min at 200 °C, the layer measured was ~ 87 nm. The XRD of the aforementioned samples were obtained which is provided in Fig. 2. The as-deposited layer has no peaks confirming the amorphous state of the deposited film, which would be considered as amorphous GST (AGST) for the rest of the paper. Few peaks can be observed in the sample annealed at 200 °C indicating the transition towards the crystallization especially FCC as peaks at ~ 24° and ~ 29° appears. On further annealing, peaks at 29° get stronger indicating more crystallization along with few smaller peaks. This is the indication of the more crystallization with higher domains which is in agreement with other investigations^66^. However, the peaks at higher temperature are not very strong or misaligned^67^,which might be due to the stress in the GST at the interface with Si. Wang et al*.*^68^ have investigated such stress between GST on few substrate and their effects on the film characteristics. To observe the crystallization temperature and process, DSC measurement of GST/Si were plotted along with the resistance *w.r.t.* temperature shown in Fig. 3. Almost complete process is exothermic with a constant rate with a clear peak at ~ 150 °C. It, also, can be observed that the crystallization process starts at ~ 144 °C and finishes at ~ 148 °C, whose peak appears at DSC curve. There is a change of approximately 2 order of change in the resistance, which is quite lower than other reported works^64^. However, the reported results are of either deposited GST on insulator or investigated in bulk/powdered form. Inset in Fig. 3 shows the resistance plot for higher temperature with no further switching of the film and representing the absence of hex structure. This phenomenon supports our assumption of the stress between Si/GST interfaces. So, for the rest of the paper, the as-deposited and samples annealed at 200 °C are used for other measurements and characterization. Also, the CGST is meant to be a sample film annealed at 200 °C.

**Figure 4:**
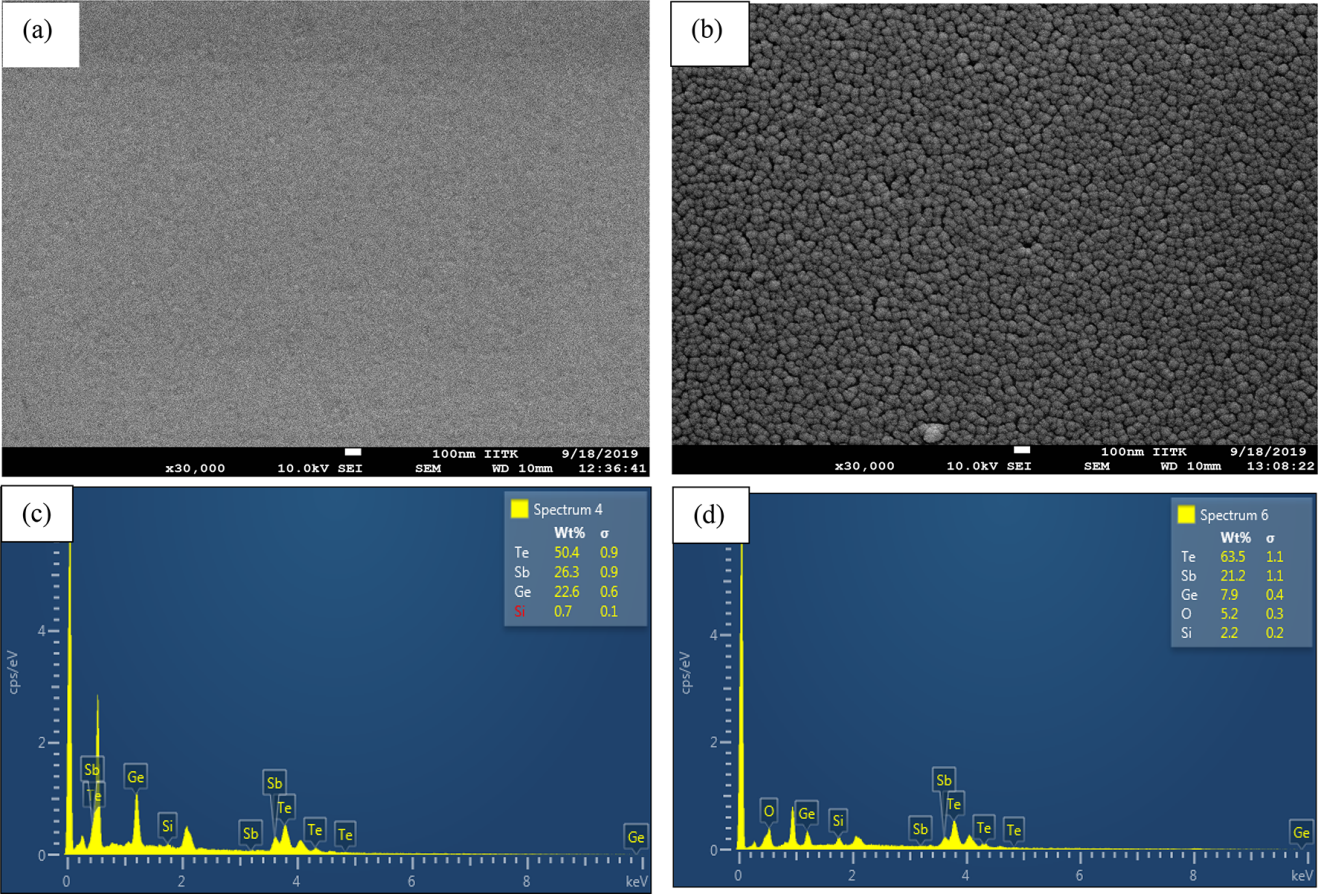
(**a**, **b**) are the SEM images, and (**c**, **d**) are the EDX of the same films of AGST and CGST respectively.

Surface morphology and its quality play an important role in deciding the device performance. The film must be dense, uniform and stoichiometric for optoelectronic devices. Morphology through SEM is shown in Fig. 4a,b at 100 nm scale for AGST and CGST respectively. Due to highly non-uniformity, AGST film is completely indistinct. On annealing the sample, the morphology exhibited smaller and denser crystallite providing higher uniformity with few *pin hole-kind* spacing between different crystallites. Figure 4c,d shows the EDX results for the respective samples. It can be observed that the weight as well as atomic percentage of Sb and Ge decreased by a significant amount whereas that of Te increased in very minute amount in atomic level. Xu et al*.*^40^ have investigated the elemental composition of GST at different temperature in detail and suggested that higher annealing temperature and time may yield the reduced Ge/Sb and increased Te. Our results are in agreement with this work. However, Si gets introduced in both phases and increased by ~ 4% in atomic scale in CGST. This might be due to the increased bonding at the interface during annealing. A little amount of ‘O’ element introduced in the CGST during annealing which may introduce the Ge–O or Te–O bonding at the surface. Apart from the intrinsic properties of PCM, these introductions of Si and O may further affect the optical and electrical properties.

**Figure 5:**
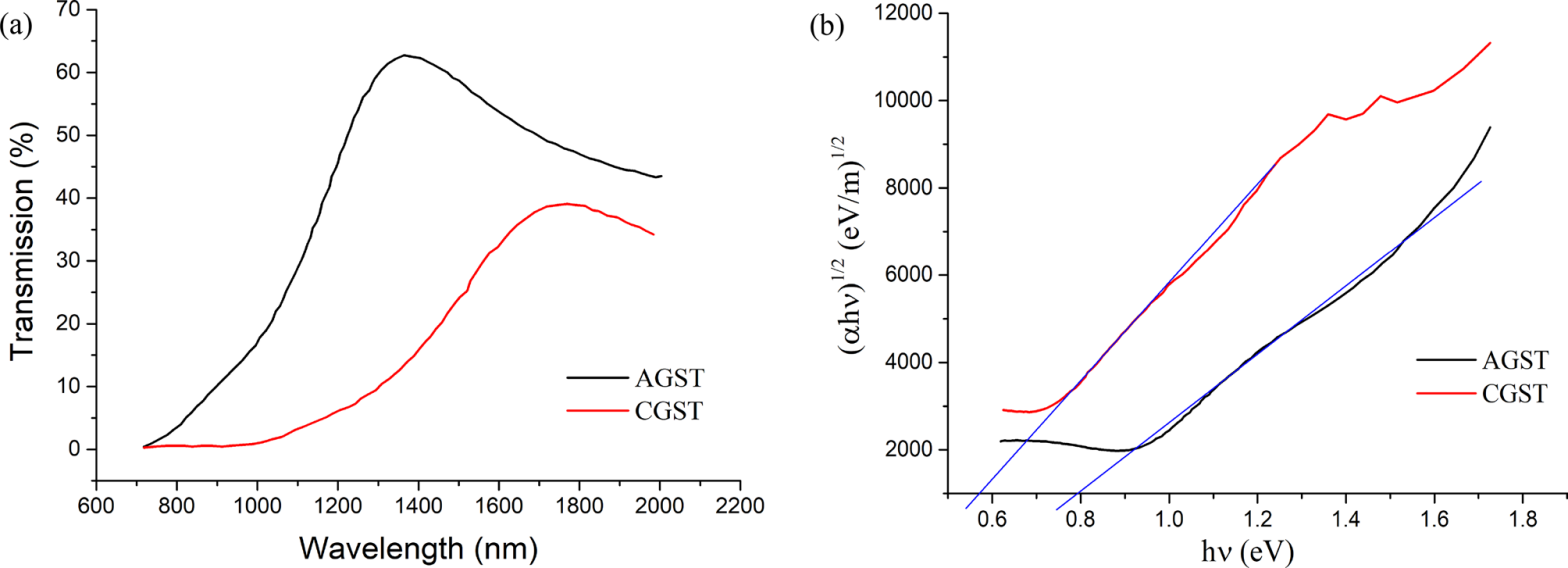
(**a**) Transmission spectrum of GST/Si stacks, (**b**) the Tauc plot for the same using data obtained in part (**a**) for bandgap estimation.

During the phase change, the optical properties of PCM modulate in terms of complex indices (*n*, *k*), where *n* represents the refractive index and *k* represents the loss. Many researchers have found that the changes in (Δ*n*, Δ*k*) and their ratio (Δ*n*/Δ*k*), treated as material's figure-of-merit (FOM)) decides many applications and scopes^69^. If the value of FOM becomes less than 1, that indicates the higher absorption or low reflection/refraction and transmission^70^. Many findings have already exploited the aforementioned phenomenon in other materials and used in different applications like modulators^71^, optical-isolators^72^, opto-electro-absorber^73^, etc. In our investigation, we found an exciting result regarding FOM which could boost further investigation of the GST/Si related research (Supplementary Information Fig. S1). In the range of 1,000–1,400 nm wavelength, optical constants changes are Δ*n* ~ 0.24 and Δ*k* ~ 0.27 whereas FOM < 1. Furthermore, in the wavelength range higher than 1,400 nm, the Δ*n* remains approximately constant with value ~ 0.2, whereas Δ*k* keeps decreasing with a value of ~ 0.05 at 2000 nm wavelength. As a result, increasing FOM at higher wavelength is obtained which might be providing quite different electrical and optical response at higher wavelength. For the optical bandgap estimation, transmission spectra were obtained using Vis–NIR spectroscopy in the range of 700 nm to 2000 nm (Fig. 5a). Using Tauc and Davis-Mott relation^74^ for the indirect transition, 0.78 eV for AGST and 0.56 eV for CGST are obtained as shown in Fig. 5b. These bandgaps and difference between them are a bit higher than in other reported works^44,45,47^. However, the widening of the bandgap difference is favourable to enhance the switching properties and higher bandgap indicates the presence of trap states, which play an important role by filling up the charge carriers to become electrically conductive^75^. In the present case, the introduction of Si and O might increase the cohesive energy and electro-negativity by forming higher energy bonds (like Ge–Si, Ge–O, Te–O) which result in the increased bandgaps^76^. Also, the slop of Tauc plot indicates the degree of disorder in the film and stability of the film. Higher the slope, lesser will be the disorder and higher stability^77^. From Fig. 5b, it can be observed that the slope for the CGST is absolutely higher than the AGST indicating the less disorder and higher stability of CGST/Si system, which is also cognate with the SEM results.

The stoichiometric changes in the film during annealing and introduction of few foreign elements can also be observed by Raman spectroscopy which is a well-known method to probe medium range of orders/stress/vibrational energy in semiconductors. Actually, photons can directly couple with the lattice vibrations and inherently reflect the local symmetry/stoichiometry. We have used 785 nm diode lasers for excitation and spotted at approximately 1 μm area. The power density on the samples was kept lower in order to avoid the radiation dependent crystallization and chosen accordingly given in^78^. Raman spectra of both phases of GST are given in Fig. S2a,b (Supplementary Information) together with the Gauss fitted peaks used to quantify the changes. The detailed analysis, related results and findings are listed in Table TI (Supplementary Information). Different normalization reference peaks were used as peak at ~ 303 cm^−1^ for AGST spectra and peaks at ~ 123 cm^−1^ used for CGST as they are the highest peak in the respective spectra.

### Electrical characterization

To observe the heterojunction behavior or rectifying properties between GST and Si, it is necessary that the metal contacts to the PCM and semiconductor must be ohmic or with negligible rectification. As GST in both of its phases exhibits p-type behavior, a low cost metal aluminum (Al) had been chosen to be deposited by thermal evaporation on both phases of GST thin film and characterized. The Al/GST shows a good ohmic contact as shown in Fig. S3a (Supplementary Information). The same experiment was also performed with Pt and Ti/Au metal, which also shows the near ohmic characteristics with GST. Al is also a preferable choice for the p-type Si. For n-Si, Al will make a rectifying contact. To make it ohmic, high-doped n-region is preferred which makes the processing complex. Many literatures have suggested for Ti/Au contact in which Ti is for adhesive as well as barrier lowering with Si and Au for low resistivity of contact^79^. We performed experiment with Ti/Au layering on n-Si using sputtering method. As deposited Ti/Au shows a highly rectifying junction, while it improves when annealed at higher temperature (shown in Supplementary Information Fig. S3b) in an ambient environment. At above 500 °C, Au/Ti/n-Si 2-terminal device shows almost ohmic behavior which can be utilized for further characterization. Overall, the structure can be further described as: Au/Ti contacts are deposited on a portion of n-Si followed by annealing. Thermally evaporated Al contacts are used with p-Si. Al, Pt and Ti/Au were characterized on the GST.

As already mentioned, the phase transition may occur due to electrically-induced heating (or Joule Heating) and optically-induced heating. We processed all electro-optical measurements under ~ μW incident power which is much lesser than mentioned phase-change optical power for GST^39^. Furthermore, we observe the electrically-induced switching in the GST/Si system which takes place at above 3 V external biasing as shown in Fig. S3 (Supplementary Information), while CGST shows no further switching up to 5 V. However, the performance of any electrical devices should be judged within smaller external biasing, further electro-optical characterizations are limited up to 3 V external biasing.

Firstly, we studied the dark current (without illumination or absence of any form of light source) of both phases of GST with both type of Si (n-Si and p-Si) and different contacts (Al, Pt and Ti/Au contacts). As already discussed, Al contact is associated with p-Si while annealed Ti/Au contact with n-Si. Contact variation is with GST only.

**Figure 6:**
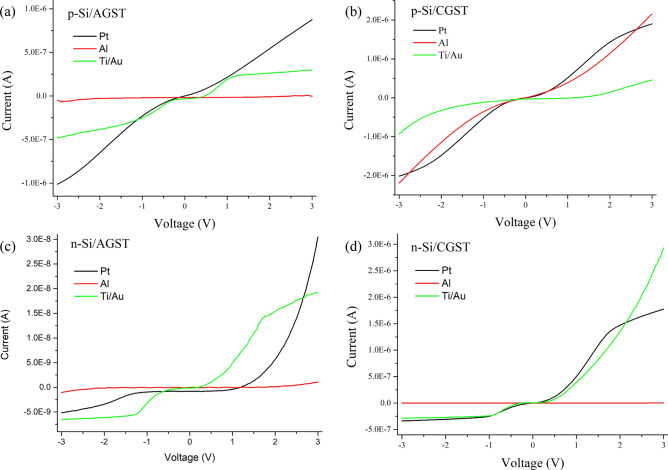
Current–voltage characteristics under dark condition for the different contacts on GST (**a**) Al/p-Si/AGST, (**b**) Al/p-Si/CGST, (**c**) Au/Ti/n-Si/AGST and (**d**) Au/Ti/n-Si/CGST.

The current–voltage characteristics of the above discussed system are shown in Fig. 6. It can be observed that p-Si make the homojunction with both form of GST, which can work in both forward and reverse biasing (Fig. 6a, b). As both of the forms exhibit p-type, the flow of current would be from majority carriers *i.e.* holes and can be collected from both sides representing the p-p homojunction. This system is not completely harnessing the characteristics of rectification and actually not promising for photodetection application in the intended range of spectrum. Similar behavior of GST has also been deduced with other planer and vertical structures in MS and MSM forms, and higher current in the range of mA is observed^49,52^. Furthermore, GST shows highly rectified characteristics with n-Si (Fig. 6c, d) representing the rectifying behavior, which should originate from n-Si/GST representing a type-II heterojunction (staggered or broken). All mentioned metal contacts to individual materials are nearly ohmic in a particular conditions. Upon phase change, its characteristics changes to Schottky with complete conductive mode (thermionic emission) of operation along with a sharp variation in electrical conductance. The increment in electrical conductivity can also be backed by bandgap deduction from AGST to CGST in Fig. 5b and to be expected due to the ordering of the lattice and obliteration of the trap centers. For photodetection application, the electron–hole pair (EHP) generation and collection mostly depends upon the absorbing material (and surface area) and built-in potential at junction (and external biasing) respectively rather on metal contacts. Hence, from now, we make an obvious choice of Au/Ti/nSi/GST/Al for further electro-optical analysis on following observations: (1) as per definitions, responsivity and detectivity (which are the major performance parameters of a photodetector and discussed later in the same section Eqs. 5 and 6), decreases as the dark current increases and the chosen system shows the lowest dark current in both phases of GST and in both positive and negative biasing; (2) considering photo-current induced remains almost same for a device, independent to type of contacts, ratio of photo-current to dark-current would be much higher in this making it suitable for broadband and lower noise-equivalent power device (if dark current considered as the main source of noise); (3) Al have lower cost and need less pre-/post-processing. Although, it must be noted that the rectification ratio (Forward-current (I_F_)/Reverse-current (I_s_)) is lowest in this system which is less required for photodetection application. At 2 V, the rectification ratio for AGST is ~ 200 and for CGST it is ~ 900. The I-V characteristics of the above mentioned fabricated devices are shown in Fig. S5 (Supplementary Information).

As per the structure of p-n junction, the behavior can be considered as thermionic emission in dark condition at room temperature (RT), therefore the thermionic emission current equation (Eq. 1) in diode can be applied and junction parameters, ideality factor and barrier height, can be estimated as,

1 $$I = I_{s} \left[ {exp\left( {\frac{qV}{{\eta kT}}} \right) - 1} \right]$$

where, *q* is the electronic charge, *V* is the external forward bias, *η* is the ideality factor, *k* is Boltzmann’s constant, *T* is temperature in Kelvin (K) and *I*_*s*_ the reverse saturation current which can be expressed as (Eq. 2):

2 $$I_{s} = AA^{*} T^{2} exp\left( {\frac{ - q\phi }{{kT}}} \right)$$

where, *A* is the effective area (~ 0.3 mm^2^), A^*^ Richardson constant defined as 4πqm^*^k^2^/*h*^3^ (m^*^ is effective mass and taken as 1.52*m*_0_ for majority carriers in GST^59^) and *φ* is the barrier height. The barrier height can be calculated from Eq. (3) as follows:

3 $$\phi = \frac{kT}{q}ln\left( {\frac{{AA^{*} T^{2} }}{{I_{s} }}} \right)$$

If neglecting the factor ‘− 1′ in Eq. (1), the ideality factor can be reformulated as:

4 $$\eta = \frac{q}{kT}\frac{1}{{\frac{{d\left( {lnI} \right)}}{dV}}}$$

That is *η* can be roughly estimated by the slope of (ln*I*) *vs. V* graph in linear region. Based on above equations, the barrier heights and ideality factors are calculated to be (0.89 eV, 0.93 eV) and (20.9, 15.3) respectively for (AGST, CGST) on Si. The large ideality factor might be due to the interface states representing higher ‘stressed’ interface leading to more trap/recombination centers, which might be helpful for photovoltaic behavior but not for photoconductive. The reduction in the *η* in CGST may represent lesser stress with substrate or ‘relaxed’ interface leading to more photoconductive nature of device. Also, the increased barrier height in CGST may represent the better rectification. It can be stated here that due to the higher *η* in both phases, the current transport mechanism may deviate from thermionic emission and transport mechanism, and may shift towards the tunneling and recombination which generally occurs at higher external biasing.

### Opto-electric characterization

According to the bandgap estimation of both phases of GST, the opto-electrical characterizations were done by irradiating the device with a TLS-300XU Xenon light source in the NIR range of 800–2000 nm wavelength at a low power of 3.38 μW. The light source intensity was also varied from ~ 1.5 μW to ~ 4.5 μW (within the instrument limit) at mid-range wavelength of 1,400 nm. It is worth to note here that absorption of Si in NIR region is negligible compared to GST^80^, hence the generation or collection are majorly due to the generation in the PCM region.

**Figure 7:**
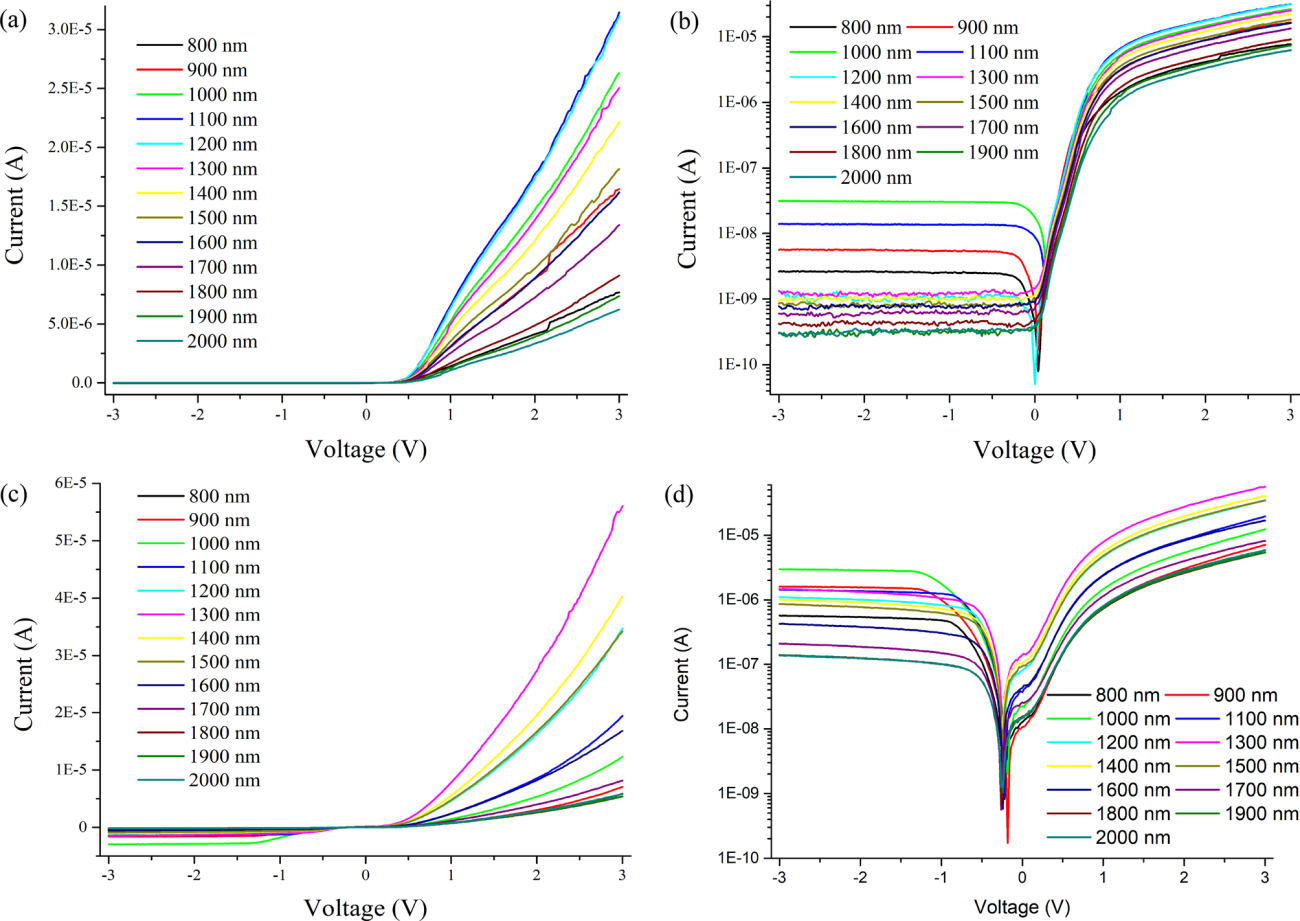
Photo-response of the device under illumination at NIR wavelength range with 3.38 μW source power (**a**, **b**) of AGST device in linear and log scale; (**c**, **d**) of CGST device in linear and log scale respectively.

Wavelength dependent experiments were performed to observe the operating region of the devices. Each wavelength has different energy; hence they will associate differently with the material lattices leading to the variation in amount of EHP generation at each wavelength. From Fig. 7, it can be observed that both phases have different interactions with each wavelength hence differing in amount of the current collection. Along with the increase in forward photo-current, the reveres saturation photo-current also increases significantly upon illumination leading to affect the rectification ratio. From Fig. 7b,d, it can be observed that the rectification ratio for AGST improves to < 900 *w.r.t.* dark condition while in CGST it reduces to ~ 500 at the corresponding forward and reverse voltage of ± 2 V. The effective EHP generation and their collection in the form of current *w.r.t.* wavelength can be better visualized by responsivity curves.

As already discussed, the performance parameters viz*.* responsivity (*R*) and detectivity (*D*) are defined as follows:

5 $$R = \frac{{I_{ph} }}{{P_{in} }}$$

6 $$D = \frac{{A^{1/2} R}}{{\sqrt {2qI_{d} } }}$$

where, *I*_*ph*_ is the photo-current (total illumination current–dark current), *P*_*in*_ is the power of incident light at particular area and *I*_*d*_ is the dark current.

**Figure 8:**
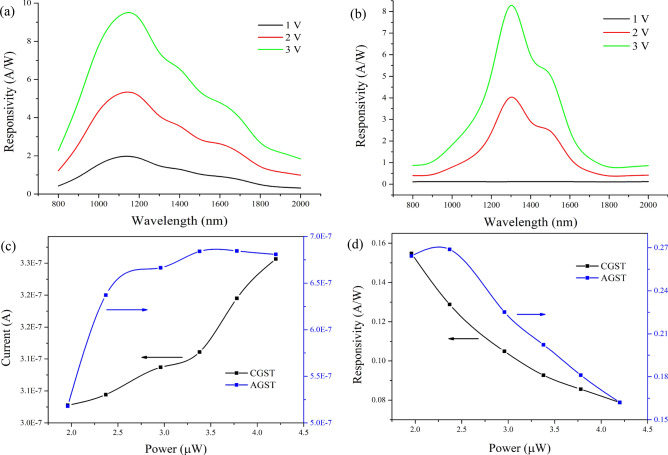
Responsivity curves at different external biasing for (**a**) AGST and (**b**) CGST based devices; (**c**) power dependence of current and (**d**) responsivity dependence on power for both phases of GST at 1 V of external biasing.

Responsivity curves are provided in the Fig. 8a,b for AGST and CGST respectively. Both of the characteristics are showing a peak at certain wavelength and the data for the maximum value of the parameters are listed in Table 1. The responsivities achieved are quite remarkable in comparison to many TMD materials based photodetectors, which are limited to 0.1 A/W at lower biasing^16,81^. This responsivity increases drastically with the increasing bias. The responsivities of AGST in entire measuring range of spectrum are higher and more stable than the CGST. It can also be observed that the range of the peak responsivity has red shift in phase transition from AGST to CGST which leads to the tuneability of device within moderate changes in mentioned performance parameters. This property can be useful in other way that if the operating temperature increases, the device performance does not change drastically. However, the major portion of the responsivity for both form of GST is higher than 0.1 A/W, hence can be used in entire NIR range. The detectivity obtained is still comparable to many planar Si/Ge or transition-metal based MSM/junction photodetectors^15^. However, this lowering in detectivity might be assumed due to a comparatively larger dark current especially in CGST. These performance parameters can be further improved by bandgap engineering, use of better fabrication pre- and post-processing, better choices of contacts, effective area optimization, stress reduction techniques etc.

**Table 1:** Performance parameters achieved for Au/Ti/n-Si/GST/Al devices.

Material	Maximum responsivity (*R*_*max*_) (A/W) @			*λ* at *R*_*max*_ (nm)	*D*_*max*_ (Jones) @
	1 V	2 V	3 V		1 V
AGST	1.95	5.25	9.3	1,100	2.3 × 10^9^
CGST	0.15	4.03	8.29	1,300	4.8 × 10^8^

We also performed the characterization by varying the source intensity of the incident light up to the available instrument’s limit. The experiments were observed at 1,400 nm wavelength light at 1 V external biasing. The characteristics are depicted in Fig. 8c, current variation, and Fig. 8d, responsivity *w.r.t.* power. From Fig. 8c, the current in CGST increases almost linearly while in AGST it shoots initially and get constant at higher power. Hence, the responsivity decreases drastically for both phases which indicates that the EHP generation and collection is not in proportional to the incident power or photo-current is not increasing significantly with increase in incident power. By these observations, we can hypothesize here that the photo-conduction (photodiode mode) are dominant in CGST. While, conduction due to photo-thermal effect may be dominant at the lower incident light power (or due to the first exposure to light) preferably in AGST and then getting to normal thermionic photo-conduction due to higher traps and recombination centers in AGST/n-Si interface. This phenomenon is also backed by the higher calculated ideality factor. Also, especially from Fig. 8d, the responsivity and similarly detectivity (as *D* is directly proportional to *R*) can be improved with lower power source. This might be due to the traps at the interface. Under a weak source power, the lower energy EHP generated may trap in the trap-centers which lead to the lower recombination and elevate the lifetimes of the photo-excited carriers, resulting in higher *R* and *D*. The same logic can be used for the higher *R* and *D* of AGST-based device than CGST-based as the traps may be higher in AGST indicated by ideality factor estimation.

### Estimation of band alignment

**Figure 9:**
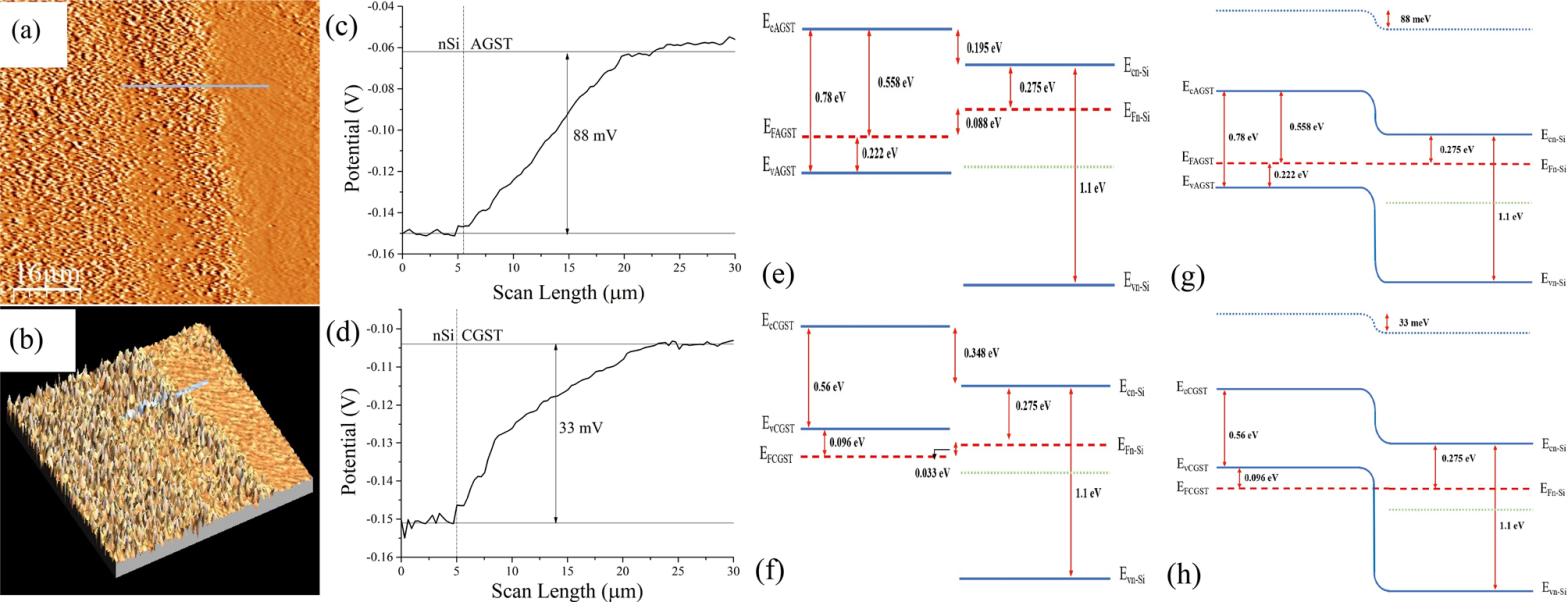
(**a**) Topography of the as-deposited GST at the n-Si interface; (**b**) 3D perspective view of the (**a**) showing the interface; (**c**,**d**) the spatially mapped surface potential at the interface along the line depicted in (**a**,**b**) measured from right to left; (**e**,**f**) represent respective band position estimation for n-Si/AGST and n-Si/CGST; (**g**,**h**) are respective band-alignment diagram at equilibrium.

To examine the band-alignment at the interface of the n-Si/GST interface, KPFM measurements were performed as shown in Fig. 9a,b. The measurement methodology and setup are adopted from Tong et al*.*^59^ and Monu et al*.*^14^. To avoid topographic artifact, dual pass taping mode was employed. The first scan was for topography and then tip was raised to 5 nm above the sample surface for work-function/surface-potential difference measurement. Here, we examine the change in surface potential of n-Si and both phases of GST across n-Si/GST depicted in Fig. 9c for n-Si/AGST and Fig. 9d for n-Si/CGST, which also provided the estimation of work-function difference and the evidence of junction formation at n-Si/GST interface for both phases of GST.

From the Fig. 9c,d, the work-function difference between n-Si and GST can be extracted as

$$\begin{aligned} & \phi_{n - Si} - \phi_{AGST} \cong - 88\,{\text{meV}} \\ & \phi_{n - Si} - \phi_{CGST} \cong - 33 \,{\text{meV}} \\ \end{aligned}$$

The work-functions of both phases of GST are observed to be greater than of n-Si; ~ 88 meV in AGST and ~ 33 meV in CGST. For Fermi-level estimation, we employed the Hall measurement to obtain the majority carrier (*p*) in both form of GST at 300 K and found to be *p* ~ 8.93 × 10^15^/cm^3^ in AGST and *p* ~ 2.27 × 10^21^/cm^3^ in CGST. The position of Fermi-level (*E*_*F*_) with respect to valance-band (*E*_*v*_) can be calculated by the Fermi–Dirac distribution function as given in Eq. (7):

7 $$p = N_{v} exp\left( {\frac{{E_{v} - E_{F} }}{kT}} \right)$$

There are still a lot of disputes on the location of *E*_*F*_ for CGST as every method of depositions/mechanisms and substrate yields in different Fermi position but it is more accepted as p-type degenerate semiconductor^45,59^. Also, from the huge carrier-concentration, we are assuming it as degenerate semiconductor and Fermi-position of CGST can be estimated by Eq. (8) given below:

8 $$p = N_{v} \frac{2}{\sqrt \pi }exp\left( {\frac{{E_{v} - E_{F} }}{kT}} \right)$$

where, *N*_*v*_ is the effective density of states at the valence band which further can be defined as *N*_*v*_ = 2(2π*m*_p_^*^kT)^3/2^/*h*^3^; *m*_p_^*^ is the effective masses of the holes and taken as 1.52*m*_0_. Based on Eqs. (7) and (8), (*E*_*v*_ − *E*_*F*_) are calculated to − 0.222 eV and + 0.094 eV for AGST and CGST respectively. From these values, it is clear that the Fermi-level of AGST (*E*_*FAGST*_) resides inside the bandgap indicating almost intrinsic semiconductor as the bandgap obtained is 0.78 eV for AGST. Whereas, the Fermi-level of CGST (*E*_*FCGST*_) is 0.094 eV below the valance-band edge (*E*_*vCGST*_) indicating the degenerate-type semiconductor. The n-Si used here are estimated by (*E*_*c*_ − *E*_*F*_) = 0.275 eV and *E*_*g*_ = 1.1 eV. Based on the above mentioned parameters, the band-alignments are estimated in Fig. 9e,g for n-Si/AGST and in Fig. 9f,h for n-Si/CGST. The band-alignment is staggered for n-Si/GST heterojunction for both phases of GST. However, n-Si/AGST is completely staggered in either biasing (forward or reverse). From Fig. 9g, the forward bias (+ ve at AGST and − ve at n-Si) will provide a large forward current while reduces the reverse current drastically in reverse bias (− ve at AGST and + ve at n-Si). Upon illumination, the EHP generated in AGST will be swapped away drastically on the opposite sides and contribute to the increase in forward-current. The explained phenomenon is also confirmed by electrical characteristics in Fig. S5, Fig. 7a,b. In the same device, the changes in reverse-current will be very less and have small increment upon illumination. In Fig. 7a,c, the reverse-current is smaller than CGST-system (Fig. 7b,d) and increment in illumination current in reverse bias is also very small. In n-Si/CGST system, the forward characteristics remain almost similar to AGST with higher current density due to higher carrier concentration. While in reverse biasing, it can be seen from Fig. 9e,g that the reverse currents are higher and are mostly due to tunneling across the junction rather than photo-conduction. Similar effect can be observed under illumination as reverse photo-current increases drastically in the n-Si/CGST system which might be due to the combination of tunneling and photo-thermal generation of excess carries. Furthermore, it can be observed that the CGST system has very small characteristics portion in second quadrant even after repetitive measurements. Though very small, still it should not be present in any semiconductor based p–n junction/heterojunction devices. This phenomenon might be due to few reasons discussed ahead. The appearance of second quadrant characteristics might be due to thermoelectricity, *i.e.* long term excitation and measurement, the temperature may have risen and likely to rearrangement of lattice structure. This phenomenon does not appear in AGST because the lattice rearrangement starts happening at comparatively larger temperature difference. While in CGST, the lattices keep rearranging themselves as we found in our case. Also, this might be due to band-misalignment while heating the sample for phase change.

## Conclusion

In summary, we demonstrated a heterojunction with the phase change material (GST) and Si stack which can be modulated in different operating spectrum. The key factor of this switching is to utilize the phase change of GST that provides a large change in optical absorption, resistivity, etc. Both phases of GST, being intrinsically p-type, are found to make a homo-junction with p-Si, while a good rectifying (type-II) junction with n-Si. Photoconductive mode along with the photo-thermal effect have been observed in the devices with good rectification. A high responsivity of greater than 9 A/W and moderate value of detectivity ~ 2 × 10^9^ Jones were obtained under moderate biasing of 3 V at low incident power of 3.38 μW. The responsivity remains higher than 0.1 A/W even for lower biasing (1 V) and for entire considered spectrum (800–2000 nm) making this device useful in NIR region. Optimized regions were obtained for both phases of device *i.e.* AGST is providing optimized output around 1,100 nm while CGST at 1,300 nm. The electrical and optical behaviors are explained through the band-alignment of n-Si/GST interface by measuring the work-function difference and carrier density which are found to be in well agreement with the opto-electric characteristics. Present investigation may provide a promising basis towards the development of high-performance CMOS/Si/SOI compatible heterojunction with improved performance parameters for future optoelectronic applications, especially for photo-detection in NIR region.

## Supplementary information

Supplementary information
