# Supplementary material for: CMOS compatible novel integration solution for broad range tunable photodetection using phase-change material based heterostructures
Source: Sci Rep. 2020 Jul 7;10:11131. doi: 10.1038/s41598-020-67950-2 (PMC7341851; doi:10.1038/s41598-020-67950-2)
Supplement: Supplementary file 1 — Supplementary information [file 41598_2020_67950_MOESM1_ESM.docx]

Supplementary Information

**CMOS compatible novel integration solution for broad range tunable photodetection using phase-change material based heterostructures**

**Vibhu Srivastava^1^, Prateek Mishra^1^, Sunny^1*^**

^1^Department of Electronics and Communication Engineering,Indian Institute of Information Technology Allahabad, Prayagraj, 211015, India

*[sunnymeharwal@gmail.com](mailto:sunnymeharwal@gmail.com)

1. Ellipsometric Analysis of refractive indices (n, k)


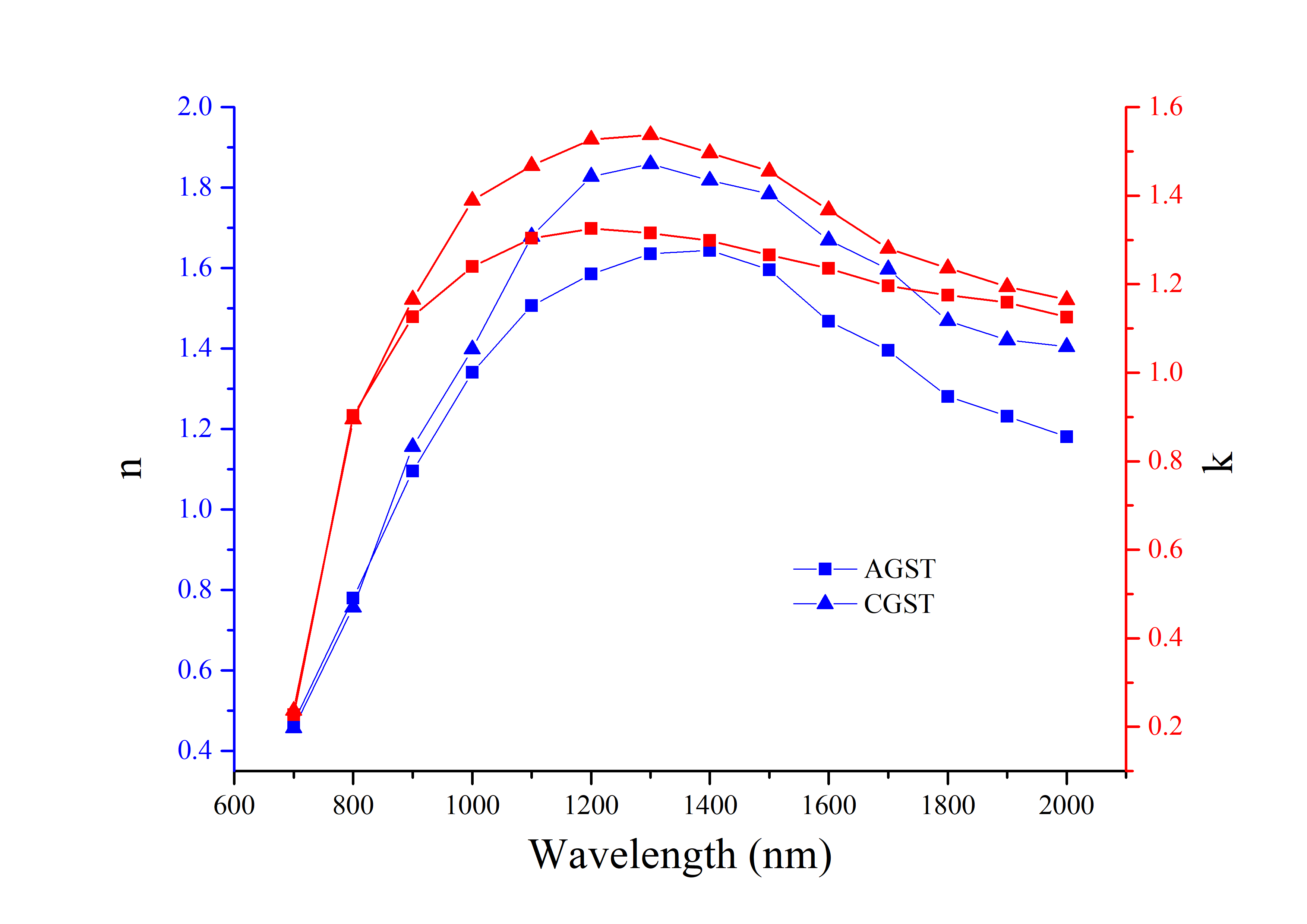


Figure S1: (n, k) parameters extracted through spectroscopic ellipsomery.

2. Raman Spectra of GST film

(a)

(b)

| 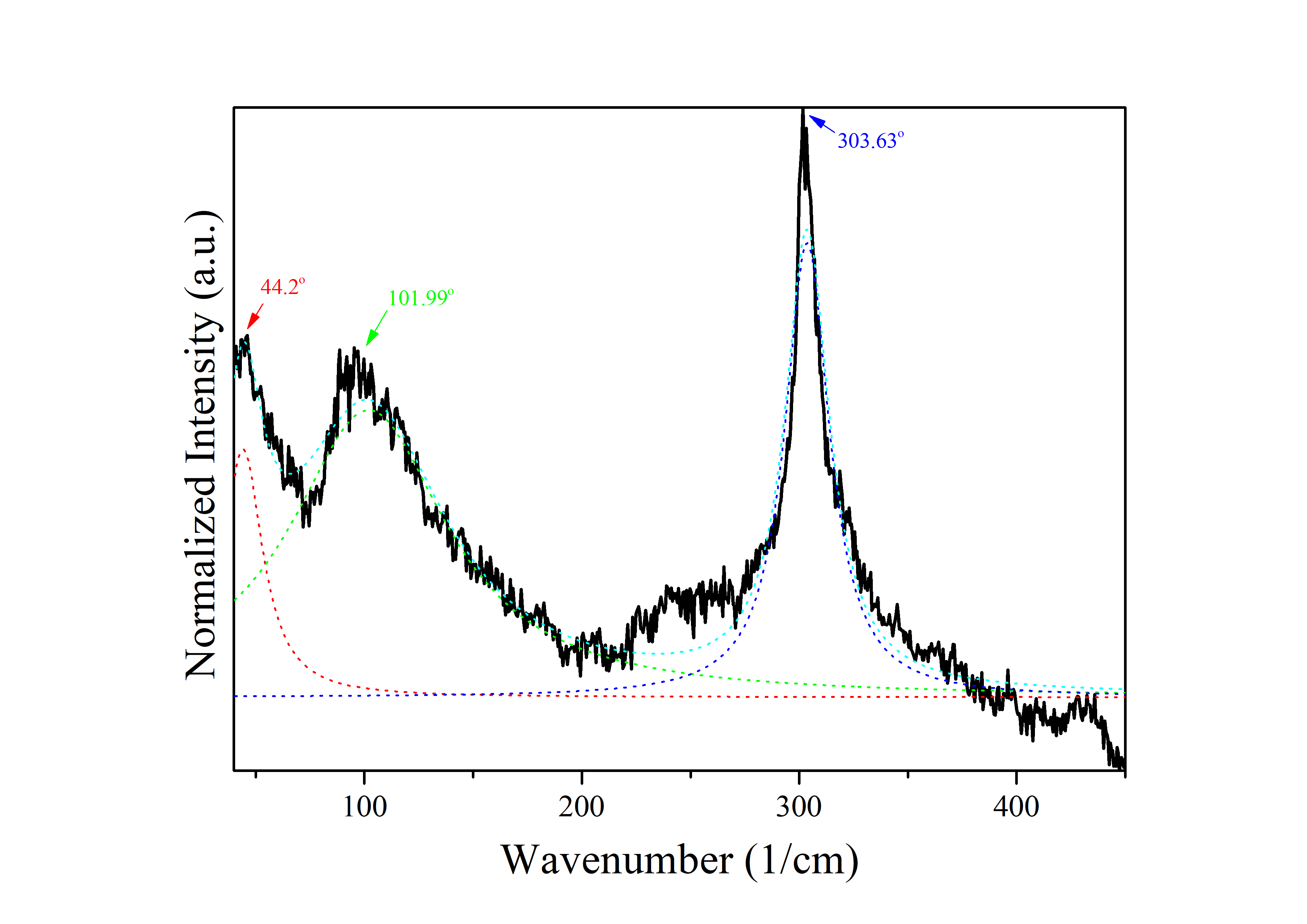 | 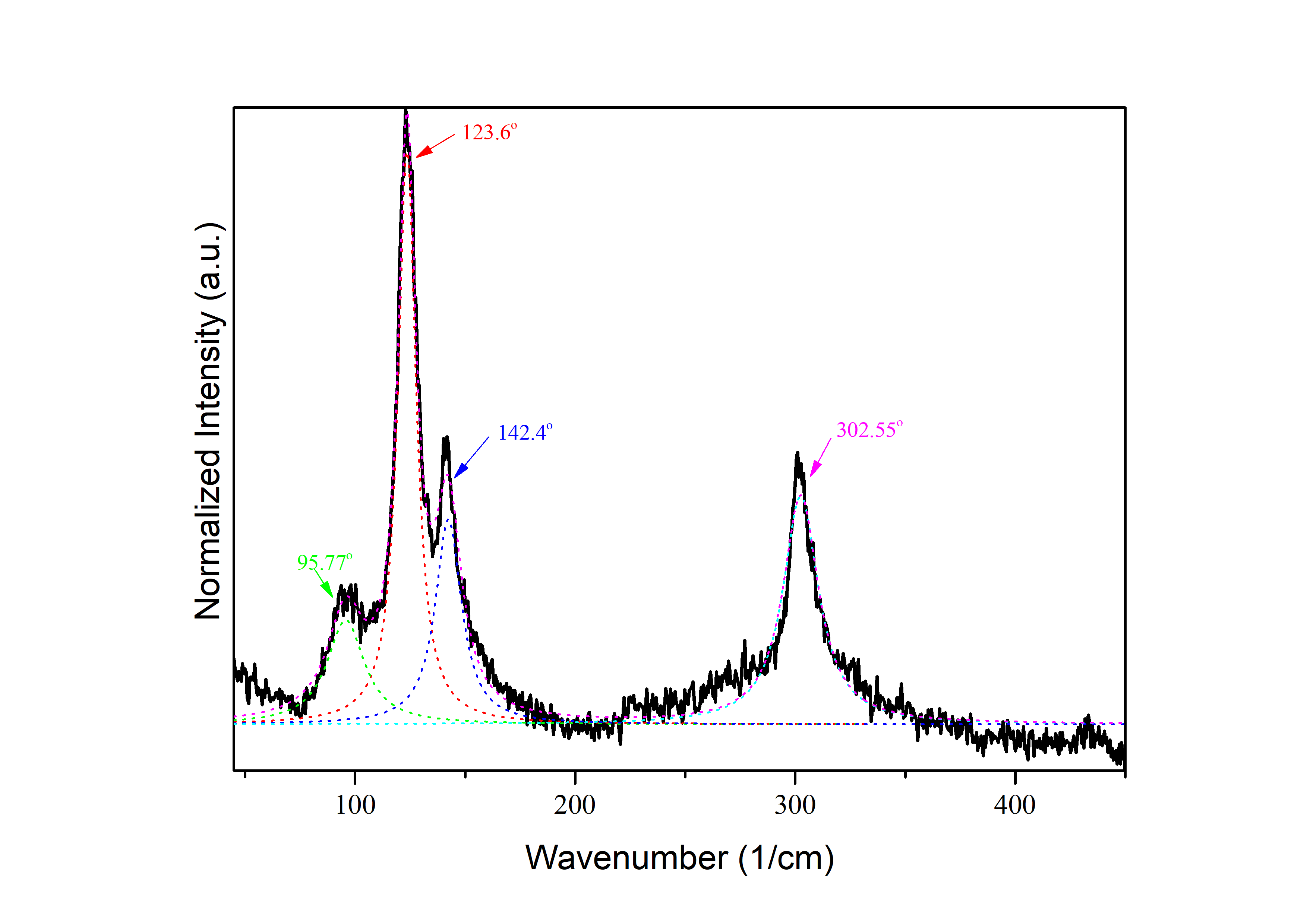 |
| --- | --- |

Figure S2: Raman spectrum of the (a) AGST and (b) CGST normalized *w.r.t.* the respective highest peaks at ~303 cm^-1^ for AGST and at ~124 cm^-1^ for CGST.

Table TI: Detailed study of Raman Spectrum

| **Inference from Raman (Lorentz Fitting, R^2^~0.96)** | | | | | | | |
| --- | --- | --- | --- | --- | --- | --- | --- |
| Material | Raman Shift/Peaks (1/cm) | Normalized Peak Intensity | FWHM | Peak Area | Peak Identity/ Inference/ Possible Materials | Speculations | Ref. |
| AGST | 44.2 | 0.374 | 21.92 | 12.88 | Boson Peak | The characterization agrees with other similar works.  The decrement in peak strength at ~301 cm^-1^ indicating the lowering of Ge and Sb content which has been seen in EDX.  An evolution of the peak at 123 cm^-1^ results an introduction of weaker bond with heavy metals which might be due to Ge-O, Te-O bonding.  Peaks around ~100 cm ^-1^ indicating the more bonding evolution of Tetrahedral GeTe_4_ or Sb_2_Te_3_ and Pyramidal SbTe_3_, which contributes more in absorption or EHP generation. | 1 |
|  | 101.99 | 0.432 | 88.15 | 59.92 | Tetrahedral GeTe_4_ or Sb_2_Te_3_. |  | 2  3 |
|  | 303.63 | 0.684 | 24.84 | 26.72 | Ge-Ge, Te-Te, Sb-Te, Sb-Sb. Responsible for crystallization and reversibility |  | 4 |
| CGST | 95.77 | 0.157 | 20.68 | 5.11 | Tetrahedral Sb_2_Te_3_, Te-O |  | 4 |
|  | 123.6 | 0.863 | 9.94 | 13.49 | Tetrahedral GeTe_4_ or Sb_2_Te_3_ and Pyramidal SbTe_3_ |  | 1 |
|  | 142.4 | 0.309 | 14.41 | 7.01 | Ge-Te, Sb-Te, Te-Te, Sb-Sb. Responsible for crystallization and reversibility |  | 5 |
|  | 302.55 | 0.344 | 19.59 | 10.58 | Ge-Ge, Si-Ge |  | 6 |

3. Ohmic contacts realization

| 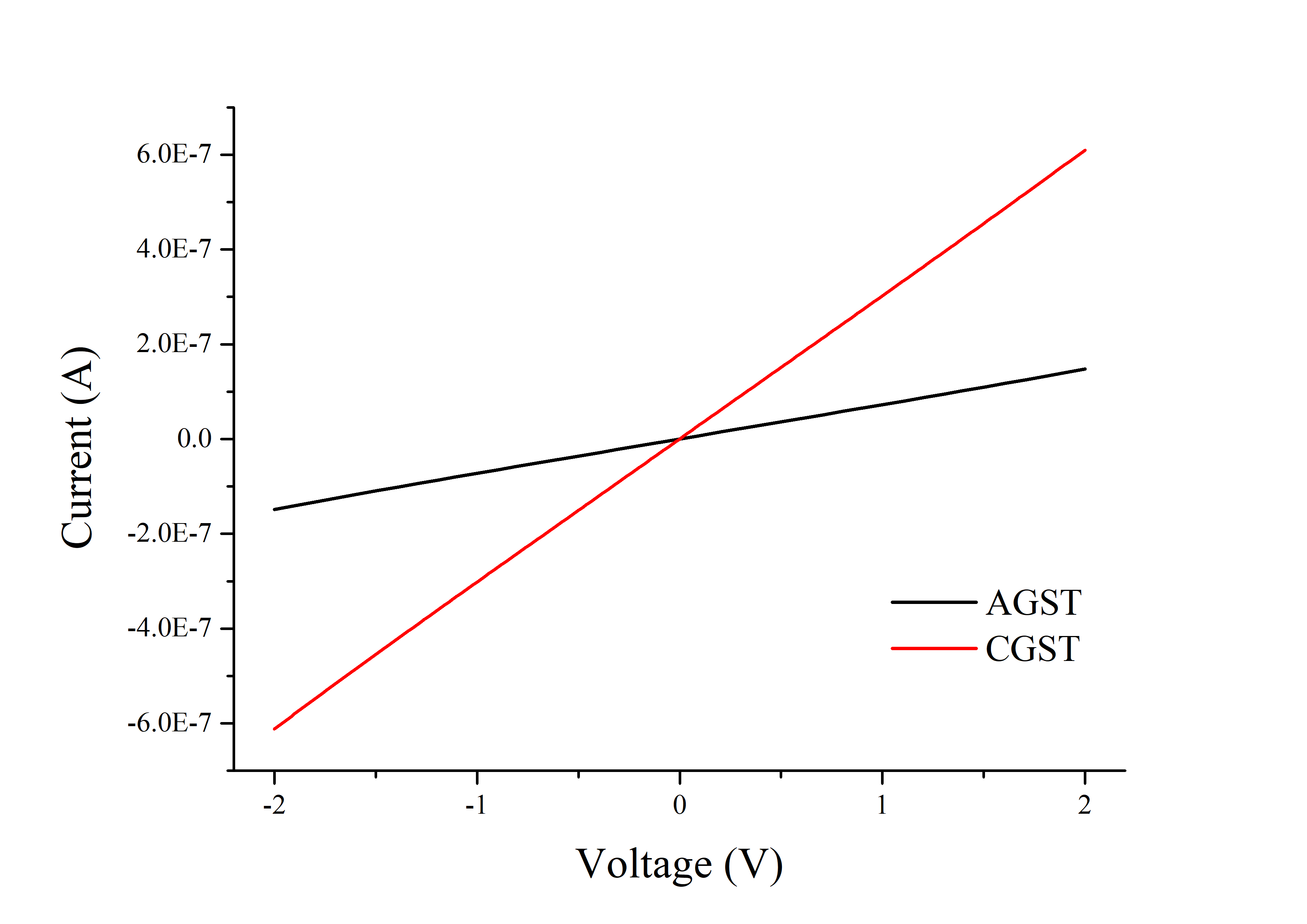  (b)  (a) | 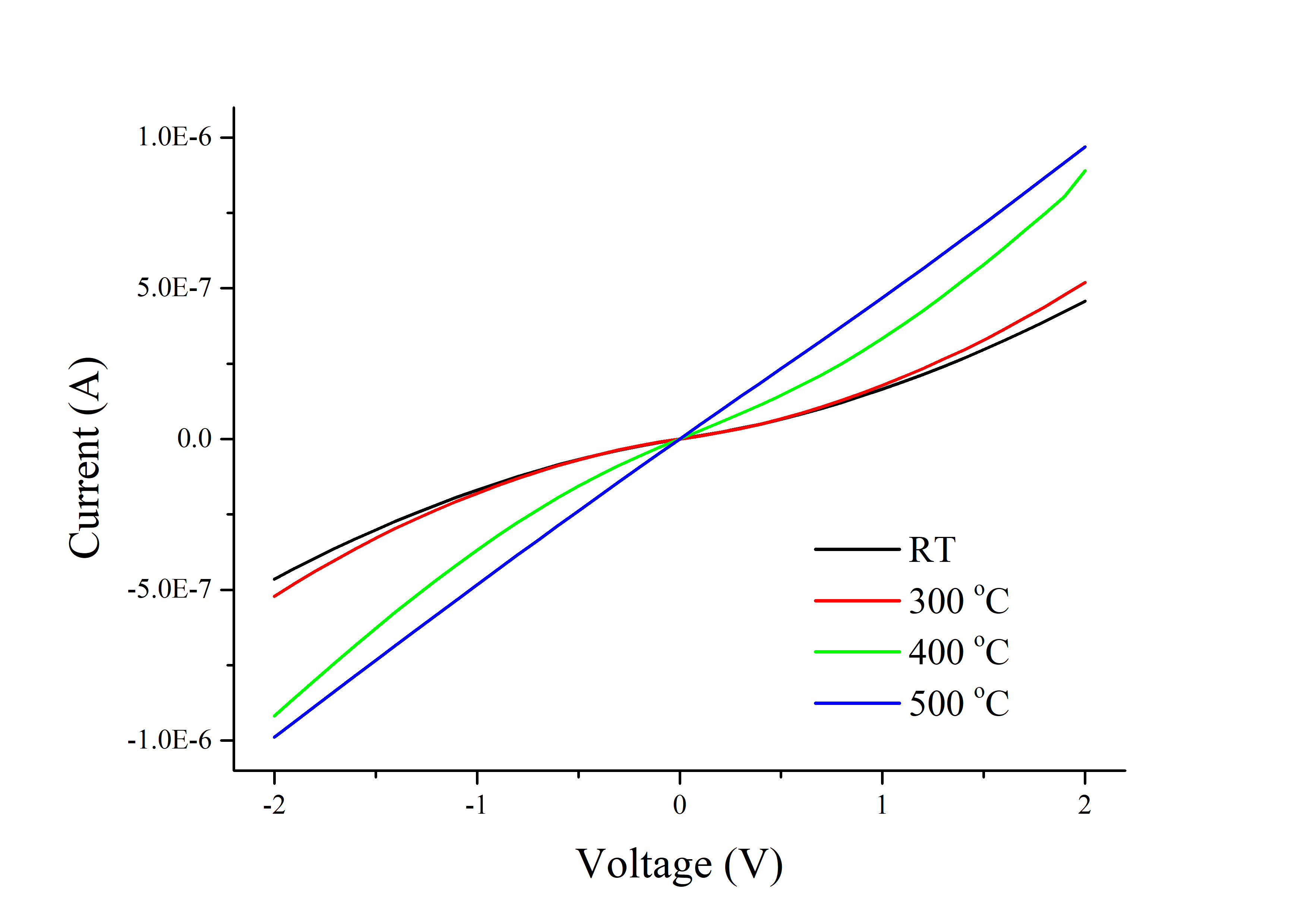 |
| --- | --- |

Figure S3: I-V characteristics of 2-terminal lateral (a) Al/GST/Al for both phases of GST and (b) Au/Ti/n-Si/Ti/Au

4. Phase Transition due to electrical excitation


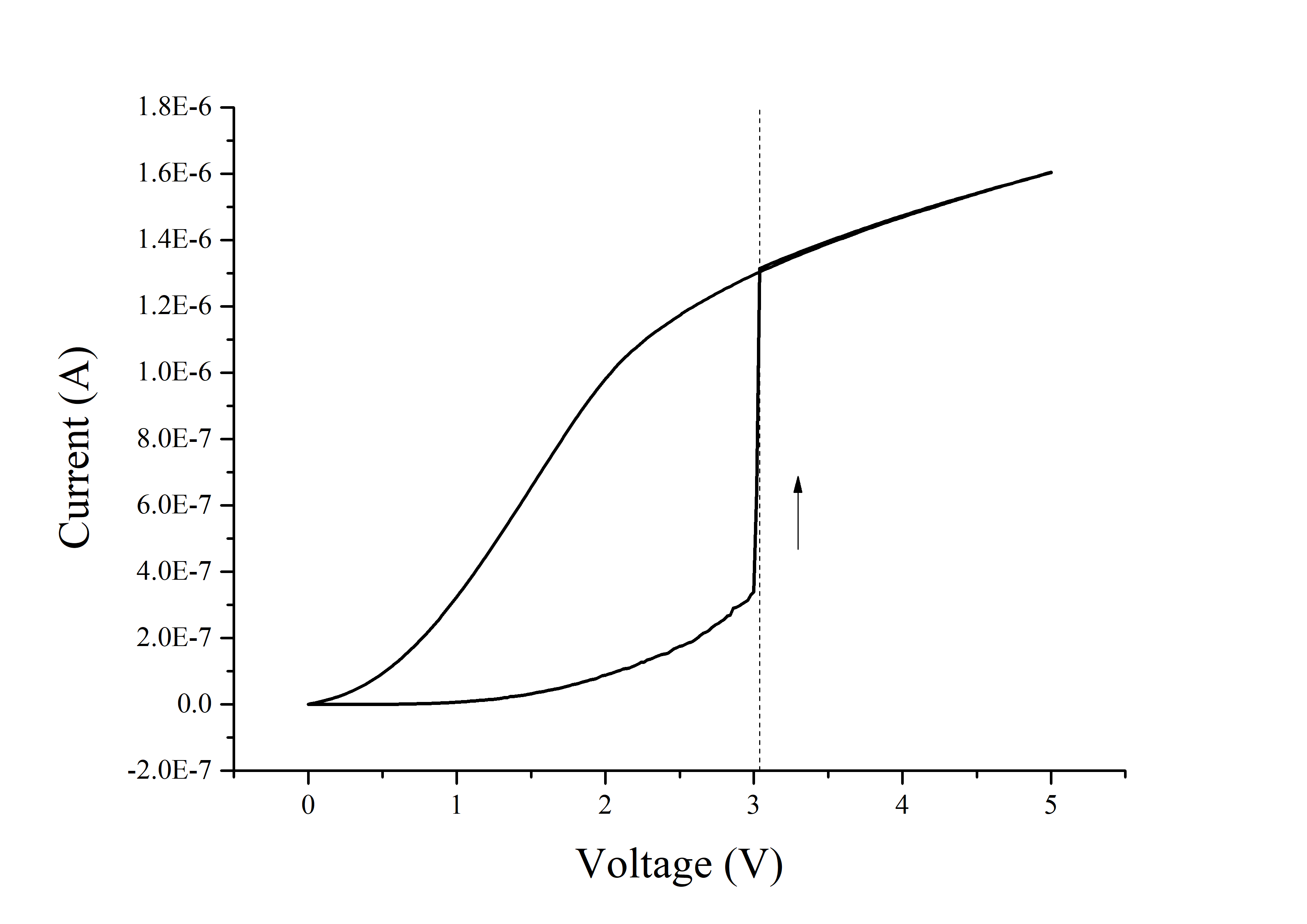


Figure S4: Typical I-V characteristics of Au/Ti/n-Si/GST/Al system. GST switches from a highly resistive state to a less resistive state at threshold bias of >3 V measured at room temperature.

5. Dark current comparison of n-Si/GST/Al system


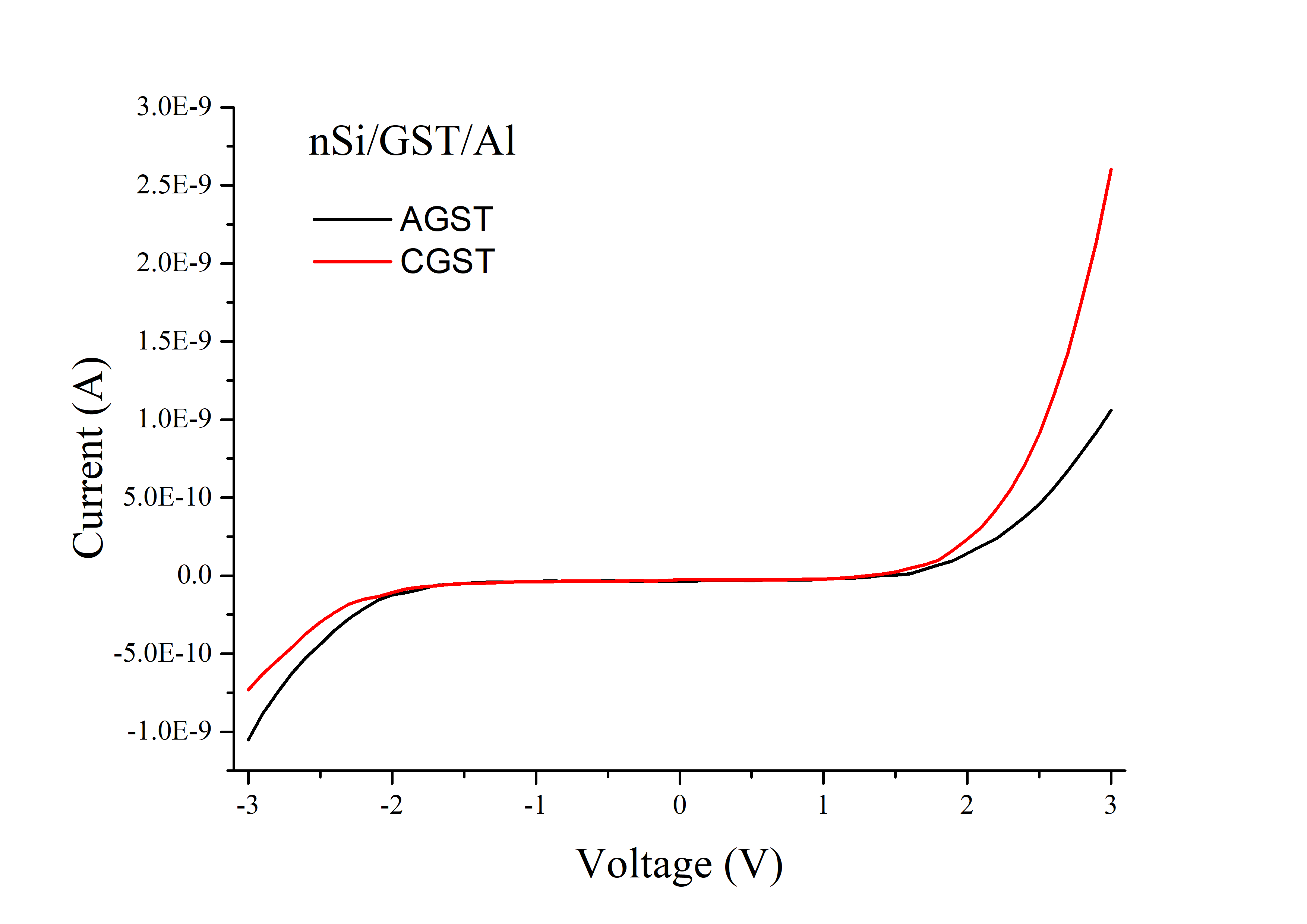


Figure S5: (Fig. 9.) Current-voltage characteristics of Au/Ti/nSi/GST/Al under dark condition

**References:**

1. Andrikopoulos, K. S., Yannopoulos, S. N., Kolobov, A. V., Fons, P. & Tominaga, J. Raman scattering study of GeTe and Ge_2_Sb_2_Te_5_ phase-change materials. *J. Phys. Chem. Solids* **68**, 1074–1078 (2007).

2. Cho, E., Yoon, S., Yoon, H. R. & Jo, W. Micro-Raman scattering studies of Ge-Sb-Te bulk crystals and nanoparticles. *J. Korean Phys. Soc.* **48**, 1616–1619 (2006).

3. Bo, L., Zhi-Tang, S., Ting, Z., Song-Lin, F. & Bomy, C. Raman spectra and XPS studies of phase changes in Ge_2_Sb_2_Te_5_ films. *Chinese Phys.* **13**, 1947–1950 (2004).

4. Kozyukhin, S., Veres, M., Nguyen, H. P., Ingram, A. & Kudoyarova, V. Structural Changes in Doped Ge_2_Sb_2_Te_5_ Thin Films Studied by Raman Spectroscopy. in *Physics Procedia* **44**, 82–90 (2013).

5. Sosso, G. C., Caravati, S., Mazzarello, R. & Bernasconi, M. Raman spectra of cubic and amorphous Ge_2_Sb_2_Te_5_ from first principles. *Phys. Rev. B - Condens. Matter Mater. Phys.* **83**, 1–8 (2011).

6. Tominaga, J. & Atoda, N. Study of the Crystallization of GeSbTe Films by Raman Spectroscopy. *Jpn. J. Appl. Phys.* **38**, L322–L323 (1999).
